# Expanding the Palette of SWIR Emitting Nanoparticles Based on Au Nanoclusters for Single‐Particle Tracking Microscopy

**DOI:** 10.1002/advs.202309267

**Published:** 2024-04-19

**Authors:** Apolline A. Simon, Lucie Haye, Abdallah Alhalabi, Quentin Gresil, Blanca Martín Muñoz, Stéphane Mornet, Andreas Reisch, Xavier Le Guével, Laurent Cognet

**Affiliations:** ^1^ Univ. Bordeaux Laboratoire Photonique Numérique et Nanosciences (LP2N) UMR 5298 Talence F‐33400 France; ^2^ Institut d'Optique Graduate School & CNRS LP2N UMR 5298 Talence F‐33400 France; ^3^ Univ. Bordeaux CNRS Bordeaux INP ICMCB UMR 5026 Pessac 33600 France; ^4^ Université de Strasbourg CNRS Laboratoire de Bioimagerie et Pathologies UMR 7021 Strasbourg F‐67000 France; ^5^ University of Grenoble Alpes Institute for Advanced Biosciences INSERM1209/CNRS‐UMR5309 Grenoble F‐38700 France; ^6^ Inserm UMR_S 1121 CNRS EMR 7003 Université de Strasbourg Biomaterials and Bioengineering Centre de Recherche en Biomédecine de Strasbourg 1 rue Eugène Boeckel Strasbourg F‐67000 France

**Keywords:** luminescence, nanoparticle, photostability, single particle tracking, SWIR imaging

## Abstract

Single‐molecule localization microscopy has proved promising to unravel the dynamics and molecular architecture of thin biological samples down to nanoscales. For applications in complex, thick biological tissues shifting single‐particle emission wavelengths to the shortwave infrared (SWIR also called NIR II) region between 900 to 2100 nm, where biological tissues are more transparent is key. To date, mainly single‐walled carbon nanotubes (SWCNTs) enable such applications, but they are inherently 1D objects. Here, 0D ultra‐small luminescent gold nanoclusters (AuNCs, <3 nm) and ≈25 nm AuNC‐loaded‐polymeric particles that can be detected at the single‐particle level in the SWIR are presented. Thanks to high brightness and excellent photostability, it is shown that the dynamics of the spherical polymeric particles can be followed at the single‐particle level in solution at video rates for minutes. We compared single particle tracking of AuNC‐loaded‐polymeric particles with that of SWCNT diffusing in agarose gels demonstrating the specificity and complementarity of diffusion properties of these SWIR‐emitting nano‐objects when exploring a complex environment. This extends the library of photostable SWIR emitting nanomaterials to 0D nano‐objects of variable size for single‐molecule localization microscopy in the second biological window, opening unprecedented possibilities for mapping the structure and dynamics of complex biological systems.

## Graphical abstract

Three families of nano‐sized SWIR probes, Au nanoclusters, polymer nanoparticles loaded with Au nanoclusters, and single‐walled carbon nanotubes are compared for their potential for high‐resolution single particle tracking. The nanoparticles and nanotubes are tracked over a long period with high precision and show an excellent complementarity for future applications in bioimaging.

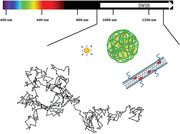

## 1 Introduction

The development of luminescent nanoprobes emitting in the second transparency biological window, where light penetration is maximized in biological tissue, is the subject of intense research for advanced imaging in biology and medical applications, allowing to observe biological processes in real and complex environments.^[^ ^1^, ^2^, ^3^, ^4^, ^5^, ^6^ ^]^ The corresponding wavelength range lies in the shortwave infrared (SWIR, also NIR II) domain (900–2100 nm), where biological autofluorescence and light scattering are minimal and significantly reduced compared to the visible and the first infrared window (NIR I, 700–900 nm).^[^ ^7^ ^]^ Although the development of currently commercially available cameras based on narrower bandgap semiconductor alloys such as InGaAs and HgCdTe enables fluorescence imaging in the SWIR, a drawback of this long wavelength range concerns the degraded resolution of optical microscopes. A powerful solution to this issue can be provided by localization microscopy approaches that have the capacity to bypass the diffraction‐limited resolution of optical microscopes. In particular, single particle tracking localization microscopy allows to interrogate molecular dynamics at the nanoscale in a variety of environments including biological specimens such as live cells or biological tissues.^[^ ^8^, ^9^ ^]^ Beyond super‐localization/‐resolution capability, the main features linked to the detection of single particles are the ability to extract minority populations behaviors and transient events that are hindered in ensemble measurements; to enable the study of dynamic behaviors in living systems; and to probe local (nano)environments (spatial, chemical, biological etc.). All these features have played a key role in the progress made over the years in understanding the intimacy of (biological) structures. Although single molecule (or particle) tracking in the NIR I has emerged,^[^ ^10^, ^11^ ^]^ its usefulness in thick samples remains limited. On the other hand, reaching the SWIR domain is challenging^[^ ^12^ ^]^ and neither dyes nor fluorescent proteins are currently suitable for single‐particle tracking in the SWIR due to their limited brightness. To date, applications of single particle tracking to biological tissues still rely on a very limited number of SWIR‐emitting nanoprobes that are bright and photostable enough to be detected through thick samples. Practically, it is mainly luminescent single‐walled carbon nanotubes (SWCNTs) that enable this type of application and have proved extremely valuable for single particle tracking (SPT) in tissues,^[^ ^12^, ^13^, ^14^ ^]^ but they are inherently 1D objects. In this context, the field would benefit from other SWIR emitting nanoparticles (NPs) having spherical morphologies to complement 1D SWCNT probes which display singular diffusion properties in crowded environments as compared to spherical ones.^[^ ^15^ ^]^ Developing SWIR NPs having distinct morphologies would indeed be key to control their accessibility in specific areas of biological tissues and so exploit the potential of SPT in thick biological samples.

A suitable single particle probe should possess the following properties: high photoluminescence (PL) brightness for super‐localization well below the emission wavelength, excellent photostability for long recording, and small dimensions for accessing restricted environments.

**Figure 1:**
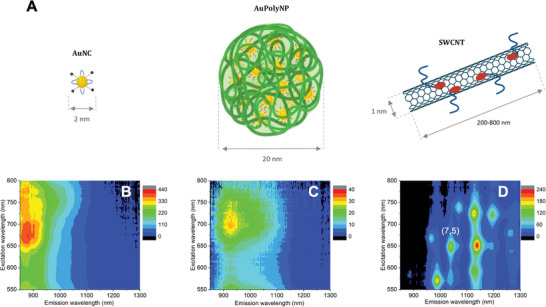
A) Scheme of the different SWIR emitters. 2D PL maps of AuNCs B), AuPolyNPs C) and SWCNTs D). The peak corresponding to the (7,5) chirality is indicated.

It was recently shown that atomically precise gold nanoclusters (AuNCs) represent a promising class of SWIR probes^[^ ^16^, ^17^, ^18^ ^]^ that bear the advantages to be ultra‐small (<3 nm), biocompatible, easy to functionalize, with tunable SWIR PL. Yet their per particle brightness and PL stability remain unknown, so the possibility of detecting them at the single particle level has yet to be elucidated. An alternative approach consists of encapsulating AuNCs at high amounts in polymers to form 0D nanoparticles.^[^ ^19^, ^20^, ^21^ ^]^ Here, we demonstrate that anisotropic surface charged AuNCs can be detected at the single particle level on 2D surfaces, and AuNC‐loaded polymer NPs (AuPolyNPs) can be detected and tracked in aqueous environments akin SWIR emitting SWCNTs, thus constituting a novel alternative for localization microscopy applications in the SWIR domain (**Figure** **1**).

## 2 Results and Discussion

Water soluble ultra‐small AuNCs surfaced‐functionalized with short dithiol‐terminated poly(ethylene glycol) molecules were chosen for their broad absorption (Figure S1, Supporting Information), high PL with quantum yield (QY) reaching 6.5% previously identified at the ensemble level (Table S1, Supporting Information). AuNCs were prepared as previously described^[^ ^17^, ^22^, ^23^ ^]^: in short, they were synthesized by a bottom‐up approach using tetrachloroauric(III) acid trihydrate (HAuCl_4_·3H_2_O) and a 3.5/0.5 molar ratio of monodentate mercaptohexanoic acid (MHA) and the bidentate ligand hexa (ethylene glycol) dithiol (HDT) in alkaline solution. This generated AuNCs having core sizes below 3 nm (according to high‐resolution Transmission Electron Microscopy (TEM), Figure S2, Supporting Information) and displaying a broad absorption range from visible to near‐infrared and corresponding emission in the SWIR from 800 to 1200 nm (Figure 1).

AuPolyNPs were prepared through nanoprecipitation.^[^ ^24^, ^25^ ^]^ For this, AuNCs stabilized by the hydrophobic ligand dodecanethiol (DDT) (Figures S3 and S4, Supporting Information) were used to make them soluble in organic solutions and insoluble in aqueous ones, which is required for efficient encapsulation upon nanoprecipitation.^[^ ^19^, ^26^, ^27^ ^]^ Here, we focused on AuPolyNPs with a small size, below 30 nm, which are more suitable for single particle tracking applications in complex environments. Indeed, studies ranging from the whole animal down to the intracellular level have shown that NPs in this size range can relatively freely move in most biological compartments.^[^ ^28^, ^29^, ^30^, ^31^ ^]^ In consequence, a poly(ethyl methacrylate) (PEMA) polymer bearing 10 mol% of methacrylic acid groups, known to reduce particle size, was chosen with an intermediate loading of AuNCs^[^ ^19^, ^29^ ^]^ (17 wt.% relative to the total mass of polymer and AuNCs). Dynamic Light Scattering (DLS) results gave a mean hydrodynamic particle size of 27 ± 2 nm. TEM micrographs showed a narrow monomodal size distribution with a mean size of 18 ± 3 nm, which is in reasonable agreement with DLS, considering that TEM gives the hard‐core size of dried NPs (Figure S5, Supporting Information). AuPolyNP optical spectra demonstrate that the optical properties of AuNCs are mostly retained when encapsulated in polymer NPs (Figure 1C; Figure S3, Supporting Information). The resulting AuPolyNPs displayed a broad absorption from <400 to 1000 nm, with bands characteristic of the encapsulated AuNCs, notably at 415 and 705 nm. They emitted in the SWIR from 900 to 1200 nm, with a PL QY of 0.65%. Though this QY is low on an absolute scale, it should be noted that it is close to that of the encapsulated AuNCs in organic solution,^[^ ^32^ ^]^ showing that encapsulation in polymer NPs is an interesting strategy to preserve the QY of AuNCs upon transfer to aqueous environments.

For comparison, we also prepared SWCNTs solutions by encapsulating raw SWCNT material in phospholipid‐polyethylene glycol (18:0 PEG5000 DSPE, Laysan Bio), a well‐known suspending agent for biological applications.^[^ ^33^, ^34^ ^]^ We chose SWCNTs synthesized by the HiPCo method (Batch 195.7, Rice University) containing a high amount of the semi‐conducting (7,5) nanotube chirality^[^ ^35^ ^]^ with the objective of using identical excitation wavelengths and intensities for AuNCs, AuPolyNPs, and SWCNTs to enable direct brightness comparisons. Indeed, (7,5) SWCNTs display strong absorption at 660 nm while emitting at ≈1025 nm, and are commonly applied in biological applications upon excitation at 660 nm.^[^ ^33^ ^]^ This makes them a fair standard for the Au‐based objects investigated in this work. We bear in mind that each type of nano‐objects displays different absorption spectra and is therefore not strictly optimally excited at the same excitation wavelengths, yet the choice of a common excitation wavelength at 660 nm is a good compromise since AuNC‐based emitters display broad absorption spectra around this wavelength.

**Figure 2:**
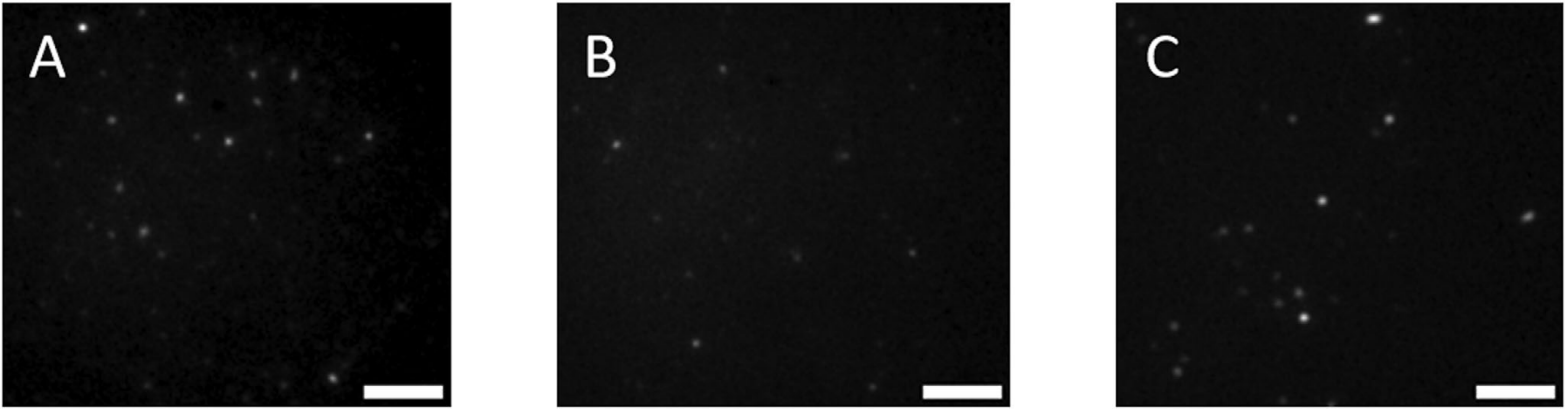
Fluorescence images of individual AuNCs A), AuPolyNPs B), SWCNTs C) immobilized on coverslips excited at 660 nm (700 W cm^−2^). AuNCs were detected in a dried state while AuNPs and SWCNTs were detected in aqueous environments. Scale bar: 5 µm.

The optical properties of AuNCs, AuPolyNPs, and SWCNTs were inspected at the single particle level using a single molecule fluorescence microscope optimized for SWIR imaging. In order, to achieve meaningful benchmarking, we performed imaging under well‐controlled conditions rather than in complex biological environments, where the influences of the composition of the solution and interactions with biological components render comparisons more difficult. **Figure** **2** shows typical images of AuNCs, AuPolyNPs, and SWCNTs dispersed on a glass‐slide and recorded with 30 ms integration time at identical excitation laser intensities.

**Figure 3:**
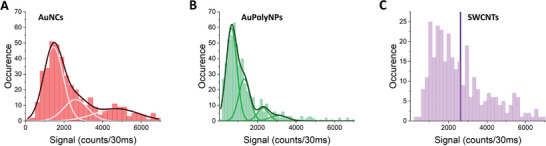
Evidence for single NP detection. Histogram of the signals for the three different samples, AuNCs (dried), AuPolyNPs (aqueous), SWCNTs (aqueous). The distributions in A and B are well‐fitted by multiple Gaussian curves. The position of the second, third, and fourth maxima were multiple of that of the first one. This observation indicates that single particles are detected in the first peak and two, and more particles are detected in the others. The histogram in C is broader with a median equal to 2700, reflecting a polydisperse distribution as expected for SWCNTs of several lengths.

We found that AuNCs could be detected as individualized entities when dried on the polylysine‐coated coverslips (but not in aqueous conditions, not shown), while AuPolyNPs and SWCNTs are easily detected both in dried and aqueous environments. Indeed, from images displayed in Figure 2, the detection of diffraction‐limited points (having Full Width at Half Maximum close to the diffraction limit given by 0,61λ/NA ≈480 nm) is the first indication that AuNCs and AuPolyNPs are detected at the single particle level. Note the presence of few elongated particles in Figure 2C as expected for SWCNTs with longer lengths than the diffraction limit. In order to confirm that the great majority of resolved discrete spots may be attributed to single NPs, we constructed the histograms of signal intensities corresponding to each spot by fitting the diffraction‐limited signals to 2D Gaussian curves having width equal to the microscope diffraction limit (see Experimental Section, **Figure** **3**).

The signal intensity histograms reveal the presence of one main population and minority subpopulations having signals that are multiples of the main population one (Figure 3A,B). This indicates that single particles correspond to the first populations while the subpopulations represent the situation of two or more particles detected within the diffraction‐limited spot. Note that for SWCNTs, the signal distribution is less monodisperse as expected by the dispersion of length of nanotube preparations (spanning typically from 200 nm up to 800 nm).

When comparing the PL of the different types of emitters in an aqueous environment, AuNCs could not be observed at the single particle level (which was expected from the per particle brightness determined in the ensemble, see Table S1, Supporting Information), yet we observed that it becomes possible to detect them at the single particle level in dried condition with PL levels of the order of that of SWCNTs. We believe this is related to previous observations having shown significant PL enhancement by surface charge injection.^[^ ^23^, ^36^ ^]^ On the other hand, AuPolyNP emission of single particles (≈650 counts/30 ms) is found to be only ≈4 times lower than that of SWCNTs (median ≈2700 counts/30 ms) that are known to be very bright emitters, bearing in mind that AuPolyNPs and SWCNTs are also very differently shaped objects. Indeed, AuPolyNPs are small spherical NPs (≈20–30 nm) in contrast to SWCNTs that are thin and long 1D objects (typically, 400 nm length, 1–3 nm diameter).

**Figure 4:**
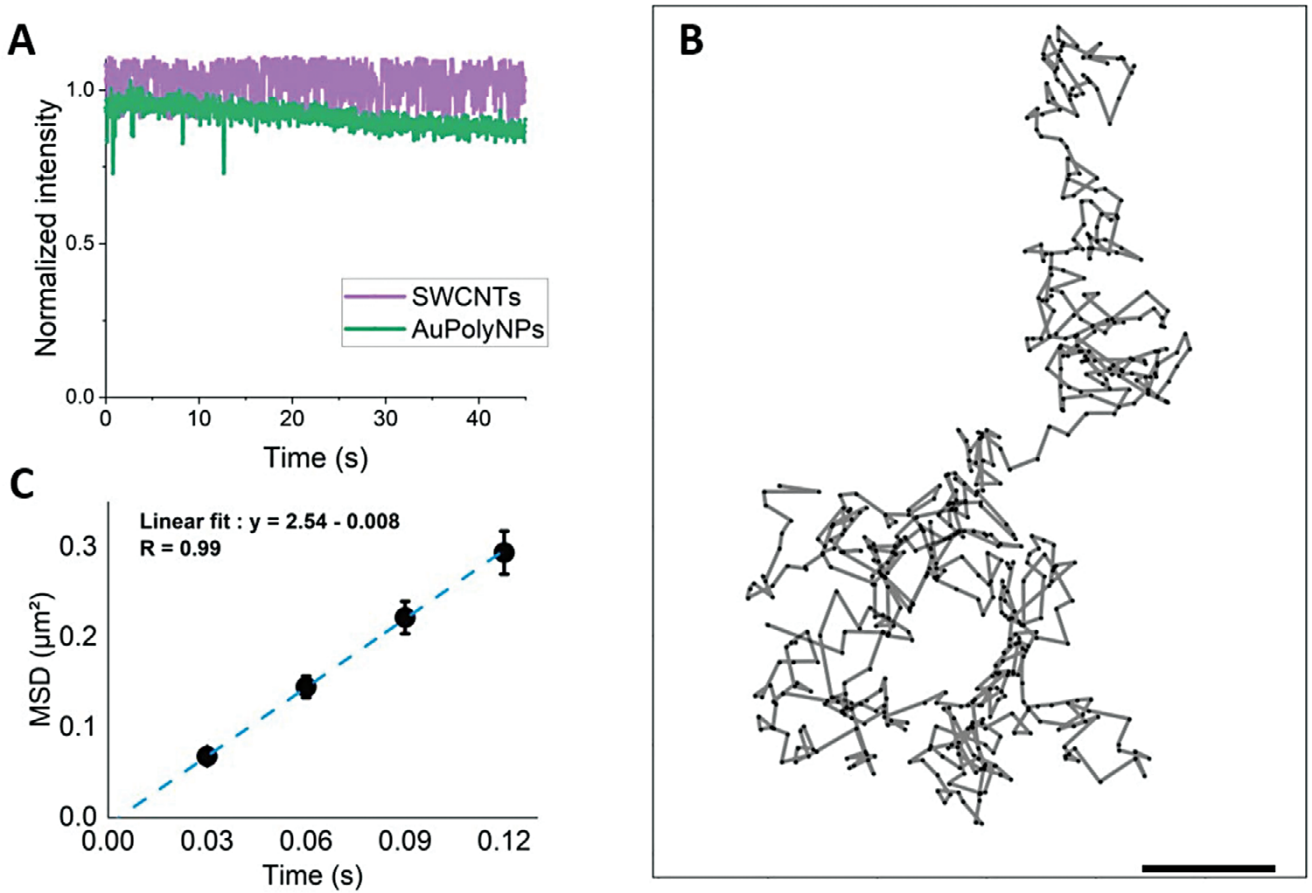
A) Photostability of a single AuPolyNC and a single SWCNT (normalized emission intensity). B) example trajectory of a single AuPolyNC recorded by SPT. Scale bar  = 1 µm. C) average MSD plot of 191 trajectories with a linear fit.

We next investigated the possibility of tracking single AuPolyNPs akin SWCNTs, which have already demonstrated great promise in biological studies due to their SWIR emission range.^[^ ^37^, ^38^ ^]^ **Figure** **4**A shows that single AuPolyNPs immobilized as in Figure 2A can be continuously detected at a high imaging rate (30 ms integration time) for tens of seconds with low photobleaching. This excellent photostability constitutes a key prerequisite for realistic SPT applications. We next imaged, localized, and tracked single AuPolyNPs freely diffusing in a water/glycerol (1:2 vol vol^−1^) solution (see Movie S1, Supporting Information). We could record and reconstruct the trajectories of individual AuPolyNPs (Figure 4B) and further analyze their diffusion characteristics. Here we analyze the 2D projection of 3D trajectories in the microscope imaging plane which, in the case of Brownian diffusion, provides a complete description of nanoparticle behavior since each dimension behaves independently. Due to the excellent photostability, trajectory lengths were only limited by the depth‐of‐focus range of the microscope and not by photobleaching. For this, we calculated the 2D average mean square displacement (MSD) of 191 AuPolyNPs trajectories (Figure 4C). The MSD is found to be linear as expected for Brownian diffusion. Interestingly, knowledge of the viscosity of the medium (0.026  ± 0.002 *Pa*.*s*)^[^ ^39^, ^40^ ^]^ allowed us to determine the diffusion constant of the particles and thus retrieve their hydrodynamic diameter from the slope of the MSD using the Stokes–Enstein equation and taking into account localization precision of this experiment (see Experimental Section). We found a value of 26  ± 3 *nm*. This average hydrodynamic diameter measured with a single molecule approach is in excellent agreement with the diameter of 27  ± 2 *nm* measured by DLS at the ensemble level.

Interestingly, we were finally able to evaluate the per particle brightness of AuPolyNPs upon excitation at 660 m and compare it with that of SWCNTs owing to the determination of the size of AuPolyNPs obtained by our SPT analysis presented above, and to the knowledge of their molar extinction and quantum yield (Table S1, Supporting Information). We found 14 000 M^−1^ cm^−1^ for AuPolyNPs to be compared to 130 000 M^−1^ cm^−1^ for (7,5) SWCNTs (Table S1, Supporting Information). This represents a brightness ratio of ≈9 in fair agreement with that obtained from our single‐particle study (≈4 fold) shown in Figure 3 given the uncertainties on the molar extinction and quantum yield data in ensemble measurements (see Supporting Information).

**Figure 5:**
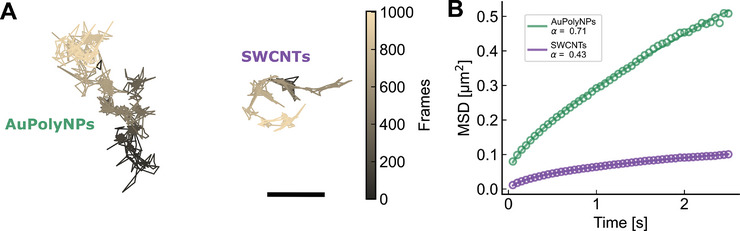
A) Example trajectories of AuPolyNPs and SWCNTs diffusing in a 3% agarose gel. Scale bar = 1 µm. B). Average MSD plots of AuPolyNPs and SWCNTs over 318 trajectories (resp. 331 trajectories). Anomalous exponent α extracted from a power law fit of the MSD curves.

Following the characterization of AuPolyNPs diffusion in an isotropic environment, we performed SPT of AuPolyNPs and SWCNTs in a 3% agarose gel as a simple model of a biological environment. Owing to their distinct morphologies, the two types of particles exhibit clearly distinct and complementary diffusion properties as can be seen on **Figure** **5**A, which depicts one representative trajectory for each NP within the agarose gel over 1000 frames (i.e., 50 s). In the trajectory of the AuPolyNP, which extends over a few microns, the particle tends to quickly explore and hop between different domains, as described previously.^[^ ^41^ ^]^ Conversely, the trajectory of the SWCNT performs less gel coverage in equivalent time, by exploring more extensively local domains due to their elongated dimensions.^[^ ^15^, ^34^ ^]^ The different diffusion features of the two types of particles can thus provide complementary information on the gel structure and diffusion environments. To highlight this multiplicity of behaviors, we calculated the mean MSD curves from a total of 318 AuPolyNP trajectories (35 556 localizations) and 331 SWCNT trajectories (72 116 localizations) on Figure 5B. The MSDs were fitted with a power law (***MSD*** (***t***) ∝ *** t*** ^***α***^, with ***α*** < 1) as commonly performed to describe anomalous diffusion. It is shown that in this complex biomimetic environment, AuPolyNPs cover more surface area per unit time than SWCNTs, with an anomalous exponent of 0.71 versus 0.43, while SWCNTs display more thorough local exploration.

## 3 Conclusion

This work demonstrates that AuNC‐based SWIR emitters constitute a promising route for expanding the palette of luminescent nanostructures for single particle tracking. Immobilized single water‐soluble AuNCs could be easily detected on the single particle level, while polymer NPs encapsulating large amounts of hydrophobic AuNCs (AuPolyNPs) could be tracked over extended periods in solution and biocompatible aqueous gels. Compared with the well‐known SWCNTs as bright SWIR emitters, these Au‐based SWIR nanostructures can exhibit comparable brightness and emission range under identical excitation conditions. This makes them particularly interesting to complement SWCNTs as diffusion probes in SPT applications to explore and reveal complex and tortuous biological structures (e.g., brain or liver tissue), as they feature very different shapes (0D vs 1D), which should enable them to access different environments. We also anticipate that advances in the design and synthesis of AuNCs and AuPolyNPs will further enhance their respective optical properties to broaden the range of applications for single‐molecule localization microscopy in the SWIR. In particular, loading of AuNCs in polymer NPs offers the possibility to tune the size of the NPs: Adapting polymer chemistry and assembly conditions allowed tailoring the size of loaded polymer nanoparticles from less than 10 nm to over 100 nm,^[^ ^29^, ^42^ ^]^ which will allow studying size‐dependent diffusion and so exploring precisely the structure of complex biological systems. At the same time, increasing the loading with AuNCs and further developments in the design of the AuNCs to achieve higher QYs, notably through optimization of the ligand sphere, should allow further optimizing their brightness for precise single‐particle tracking applications deep inside complex biological tissues. Analysis of the biological response to similar particles showed that these did not cause any adverse effect,^[^ ^43^ ^]^ opening the way for applications in complex biological systems. More generally, this study should contribute to the development of novel contrast agents for biological and medical imaging. We further believe that it will also stimulate the development of different formulations used in nanomedicine (biocompatible polymer nanoparticles, liposomes, solid lipid nanoparticles, etc.) loaded with high amounts of SWIR emitting AuNCs to enhance their optical properties and to monitor their access and fate in biological tissues.

## 4 Experimental Section

### AuPolyNPs Preparation

Poly(ethyl methacrylate) (PEMA) bearing 10 mol% of methacrylic acid was synthesized through free radical polymerization as described previously.^[^ ^19^ ^]^ Stock solutions of PEMA were prepared at a concentration of 10 g L^−1^ in acetonitrile. These solutions were diluted to 2 g L^−1^ in acetonitrile, with 17 wt.% of AuNCs (relative to the total mass of polymer and AuNC). These solutions were quickly added to a 9‐fold volume excess of phosphate buffer (20 mm, pH 7.4) under shaking (Thermomixer comfort, Eppendorf, 1050 rpm at 21 °C), followed by further dilution to the desired concentration.

### Microscopy Setup

The single‐molecule fluorescence microscope operating in the SWIR domain was built around a conventional widefield microscope equipped with a 60x/1.27NA Water immersion objective (Nikon) having high transmission in the SWIR. The sample was illuminated at 660 nm (Obis laser, Coherent) at 0.7 kW cm^−2^ by reflecting the laser on a 900 nm long pass dichroic mirror (FF875‐Di01, Semrock). For AuNCs and AuNPs a 900 nm long pass emission filter (ET900LP, Chroma) was inserted in front of a low‐noise SWIR InGaAs camera (Ninox 2, Raptor Photonics) to record single particle images. For SWCNTs, the combination of a 1000 nm long pass filter and a 1050 nm short pass filter (Edmund Optics) allowed to select resonantly excited (7,5) SWCNTs among the other SWCNT chiralities present in the HiPCo sample (see Figure 1D).

### Sample Preparation of Immobilized Particles

AuNCs, AuPolyNPs or SWCNTs we immobilized them on a glass coverslip functionalized with polylysine. More precisely, a drop of polylysine (0.01 wt.% in DI water) was placed on a coverslip at room temperature, after rinsing, and let to dry for 1 h.  The dispersion of NPs was then placed on the coverslip for another hour, followed by several rinsing. The coverslip was then mounted on the microscope for optical studies.

### Single Particle Brightness Analysis

For each NP type, 40 fields of view were acquired with identical excitation laser intensity at 660 nm on the same optical setup (except for the combination of filters, see above). Each field of view contained optically resolved emission spots as exemplified on Figure 2. For analysis, first Fidji was used to rescale the image pixel sizes to 237 nm (in the object plane). All images, then analyzed with a home‐made Matlab program where each diffraction‐limited spot was adjusted by a 2D Gaussian fit which provided the brightness of the spots displayed in Figure 3 defined as the integrated signal under the 2D Gaussian fits.

### Particle Size Determination via Diffusion Analysis

AuPolyNPs or SWCNTs were added in a 2:1 v v^−1^ glycerol–water mixture. A drop of this solution was sandwiched between two coverslips and sealed by vacuum grease before placing them onto the fluorescence microscope. Movies of diffusing particles were then acquired with a 30 ms exposure time (t_E_). Single particle tracking analysis was performed using python homemade codes.^[^ ^44^ ^]^ From the reconstructed trajectories, the Mean Square Displacement (MSD) was then computed, and the diffusion coefficients of each trajectory (*D*) were extracted using the following formulae: $MSD\;\left(t\right)=\;\varepsilon -\frac{4}{3}Dt_{E}+4Dt$, where ε is related to the localization uncertainty for a static particle  (σ) by^[^ ^45^ ^]^ ε  = σ^2^. Figure 3C displays the average *MSD* obtained from 191 trajectories. Fitting experimental data with a linear curve provided  *D*  =  0.63 µ*m* ^2^/*s*. The offset of the fit contains two terms: static localization uncertainty and a diffusion term during the acquisition of an image. Knowledge of *D* allowed to determine the static localization and we found σ  =  61 *nm*. From the value of the diffusion constant D, the hydrodynamic particle radius (r) could be calculated with the Stokes‐Einstein relation $D\;=\;\frac{k_{B\;}T}{6\pi \eta r}$, where *k*_*B*_is the Boltzmann coefficient, *T* the temperature and η the dynamic viscosity of the mixture (Pa.s)

### SPT in Agarose Gels

AuPolyNP and SWCNT water diluted solutions were added (1/20 v v^−1^) into low melting point agarose gels (3% w w^−1^, Thermo Scientific). Before mixing the solutions, the agarose was heated up using the microwave, followed by a 10 min wait for cooling. Immediately after, ≈100 µL of the final mixed solution was placed on the coverslip followed by a 5 min resting time for gel solidification before SPT recording under the microscope. Diffusing particles were recorded in movies from 2000 to 5000 frames with an exposure time of 40 ms.

## Conflict of Interest

The authors declare no conflict of interest.

## Supporting information

Supporting Information

Supplemental Movie 1

## Data Availability

The data that support the findings of this study are available from the corresponding author upon reasonable request.
