# Supplementary material for: Expanding the Palette of SWIR Emitting Nanoparticles Based on Au Nanoclusters for Single‐Particle Tracking Microscopy
Source: Adv Sci (Weinh). 2024 Apr 19;11(24):2309267. doi: 10.1002/advs.202309267 (PMC11199965; doi:10.1002/advs.202309267)
Supplement: Supplementary file 1 — Supporting Information [file ADVS-11-2309267-s001.pdf]

ADVANCED SCIENCE
Open Access

Supporting Information

for *Adv. Sci.*, DOI 10.1002/advs.202309267

Expanding the Palette of SWIR Emitting Nanoparticles Based on Au Nanoclusters for Single-Particle Tracking Microscopy

Apolline A. Simon, Lucie Haye, Abdallah Alhalabi, Quentin Gresil, Blanca Martín Muñoz, Stéphane Mornet, Andreas Reisch, Xavier Le Guével* and Laurent Cognet**

Supporting Information

Expanding the palette of SWIR emitting nanoparticles based on Au nanoclusters for single-particle tracking microscopy

Apolline A. Simon[+], Lucie Haye[+], Abdallah Alhalabi, Quentin Gresil, Blanca Martín Muñoz, Stéphane Mornet, Andreas Reisch, Xavier Le Guével* and Laurent Cognet*.*

[+] equal contribution

Dynamic Light Scattering (DLS):

The size of the obtained NPs was measured on a Zetasizer Nano series ZSP (Malvern Instruments). Each sample was measured 10 times with a run length of 10 s each. Intensity-averaged values were used. Mean values give the average over at least three independent preparations, error bars correspond to standard error of the mean.

Transmission electron microscopy (TEM):

Solutions of AuNC loaded NPs (5 μL) were deposited onto carbon-coated copper−rhodium electron microscopy grids following amylamine glow-discharge. They were then treated for 20 s with a 2% uranyl acetate solution for staining. The obtained grids were observed using a Tecnai F20 Twin transmission electron microscope (FEI Eindhoven Holland) operating at a voltage of 200 kV. Images (2,048 pixels × 2,048 pixels) were recorded using a US1000 camera (Gatan) and analyzed using the Fiji software. At least 200 particles per condition were analysed.

Estimation of the number of hydrophobic AuNCs per AuPolyNP:

The volume of one NP (V_{NP}) was calculated based on the mean NP diameter. The NP density (ρ_{NP}) was then determined using a mean density based on the loading of hydrophobic AuNCs inside the particle (L expressed as weight fraction relative to the total mass of polymer and Au) and the densities of Au ($\rho_{AuNC} = 19{,}300\ kg.m^3$) and of the polymer ($\rho_{PEMA} = 1110\ kg.m^3$):

$$\rho_{NP} = \frac{1}{\frac{1-L}{\rho_{PEMA}} + \frac{L}{\rho_{AuNC}}} \qquad (1)$$

The mass of AuNCs per particle (m_{AuNC}) was calculated using:

$$m_{AuNC} = \rho_{NP} \times V_{NP} \times L \qquad (2)$$

The number of AuNC per NP (N_{AuNC}) was calculated by taking into account the molar mass of the cluster ($M_{AuNC} = 5{,}000\ g.mol^{-1}$ with respect to Au, with $N_A$the Avogadro constant):

$$N_{AuNC} = \frac{m_{AuNC} \times N_A}{M_{AuNC}} \qquad (3)$$

Uncertainties on the obtained values originate from the size measurement and the used density of the polymer-AuNC nanohybrid but are difficult to estimate.

Spectroscopic properties

Absorption spectra were recorded on a Cary 5000 Scan ultraviolet−visible spectrophotometer (Varian). Photoluminescence (PL) spectra were recorded on a WP NIR1 spectrometer (Wasatch Photonics) using lasers at 730 nm from LCC Oxxius or at 808 nm from Cobolt as excitation sources

Quantum Yields:

*QY*s were determined from the absorbance values at the excitation wavelength ($A_{x,808nm}$) and the integral over the whole emission range (F_x) using a simplified relative method with *IR1061* in dichloromethane as reference ($QY_{IR1061} = 0.0041$, absorbances below 0.1), according to the formula:

$$QY_{NP} = QY_{IR1061}\ \frac{F_{NP} \times A_{NP,808nm} \times n_{H2O}^2}{F_{IR1061} \times A_{IR1061,808nm} \times n_{DCM}^2}$$

where “NP” corresponds to solutions of the NPs in deuterated water, “IR1061” to the reference in dichloromethane (DCM), and *n* to the respective refractive indices of the solvents (1.33 for water and 1.42 for DCM).

Uncertainties on QY measurements are supposed to be due to the limits of the relative method of QY determination. In particular, the absorption spectra of the reference and the AuNCs have very different shapes, and the emission spectra do not match perfectly. In addition, the QY

values differ by more than one order of magnitude between the two. We therefore estimate that the error on the QY can easily reach a factor of two.

Ensemble Brightness:

The brightness B is the product of the extinction coefficient of AuNC, ε_M, the number of AuNCs per NP (N_{AuNC}), and the QY of the NP (QY_{NP}):

$$B = N_{AuNC} \times \varepsilon_M \times QY_{NP}$$

The specific extinction coefficient ε_M was determined through absorbance measurements with solutions of known concentration of AuNCs encapsulated in polymer NPs to be approximately $8{,}700\ M^{-1}cm^{-1}$ at 660 nm.

For SWCNTs, the brightness B is the product of the extinction coefficient per C atom ε_{SWCNT}, the number of carbon atoms in the nanotube length (N_C) and the QY of the SWCNTs in a given surfactant (QY_{SWCNT}):

$$B = N_C \times \varepsilon_{SWCNT} \times QY_{SWCNT}$$

Table S1. Characteristics of the different NPs used in this study.

Nanoparticle type	ε per NP elemental constituent [$M^{-1}.cm^{-1}$] a)	Size [nm]	*N:* Number of AuNC/C per NP	*ε*N:* extinction coefficient per NP [$M^{-1}.cm^{-1}$] a)	Quantum yield [%]	Brightness [$M^{-1}.cm^{-1}$] a)
AuNCs b)	4,050	2-3	1	4,050	6.5	263
AuPolyNPs	10,000 per AuNC	26 (diameter)	240	2.1E+6	0.65	1.4E+04
(7,5) SWCNTs	1,600 per C atom c)	400 (length) d)	39,000	6,5E+7	0.21 e)	1.3E+05

a) excitation at 660 nm; b) in water from reference[1]; c) from reference[2] for (7,5) nanotubes at their S_{22} transition (660nm) d) as a mean length value e) derived from reference[3] after correction due to the use in this study of phospholipid-PEG as a biocompatible surfactant for SWCNTs instead of sodium cholate used in reference[3] which generates brighter nanotubes.

Comments on the uncertainties in the brightness values presented in Table S1

Comparison of such different types of nano-objects in terms of brightness requires careful consideration of the number of possible uncertainties impacting their values. For example, in the case of the AuPolyNPs sources of uncertainty include: (i) the calculation of the number of hydrophobic AuNCs (AuDDT) per NP is based on the volume of the NPs. In consequence, small differences in the estimated diameter can lead to a quite large uncertainty in the number of hydrophobic AuNCs per particle. (ii) the NP density is used to calculate its mass, which most probably varies strongly with loading in view of the strong difference in density of Au and polymer. Here a linear approach is used to estimate the density of the AuNC-polymer nanohybrids. As the number of hydrophobic AuNCs is directly related to the brightness, we estimate that the associated uncertainties can easily reach (or even exceed) a factor of two. (iii) reliable QY references for the emission wavelength range are rare, resulting likely in a mismatch between the absorbance and emission of the studied probe and the reference. We estimate that this can contribute another factor of two to the uncertainty of the brightness. (iv) other sources of uncertainty can be related to differences in the absorbance or emission behavior of the hydrophobic AuNCs upon encapsulation in polymer NPs and to scattering.

Concerning SWCNTs, uncertainties mainly originate from the still debated exact values of their QYs, as well as the mean nanotube lengths (thus carbon atom number) to consider in the calculation.

The brightness figures presented in the table should thus be considered carefully given these large sources of uncertainties.

Supplementary Figures

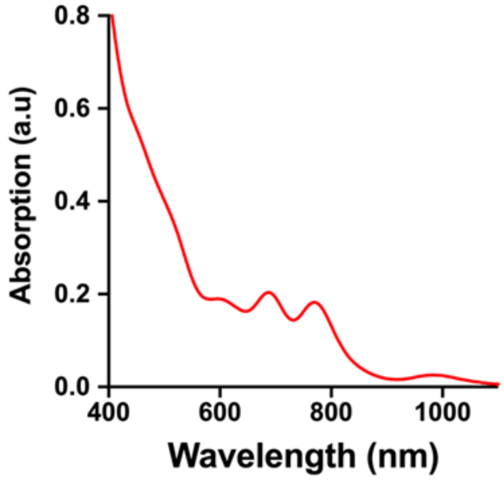

Figure S1. Absorption spectrum of AuNCs in water between 400 and 1100 nm.

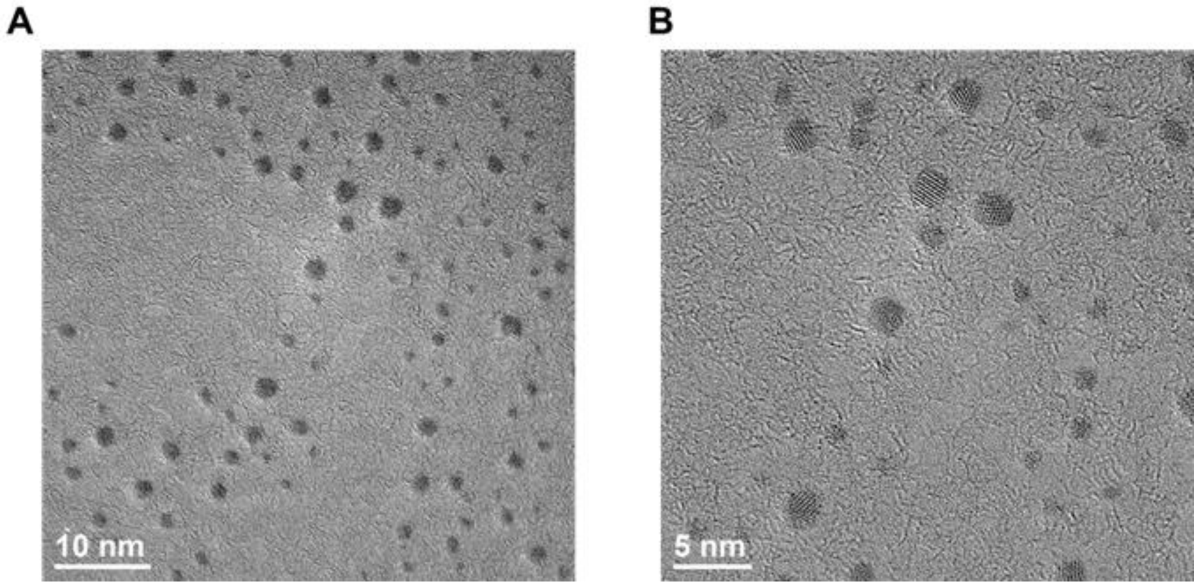

Figure S2. TEM images of hydrophilic AuNCs at two different magnifications.

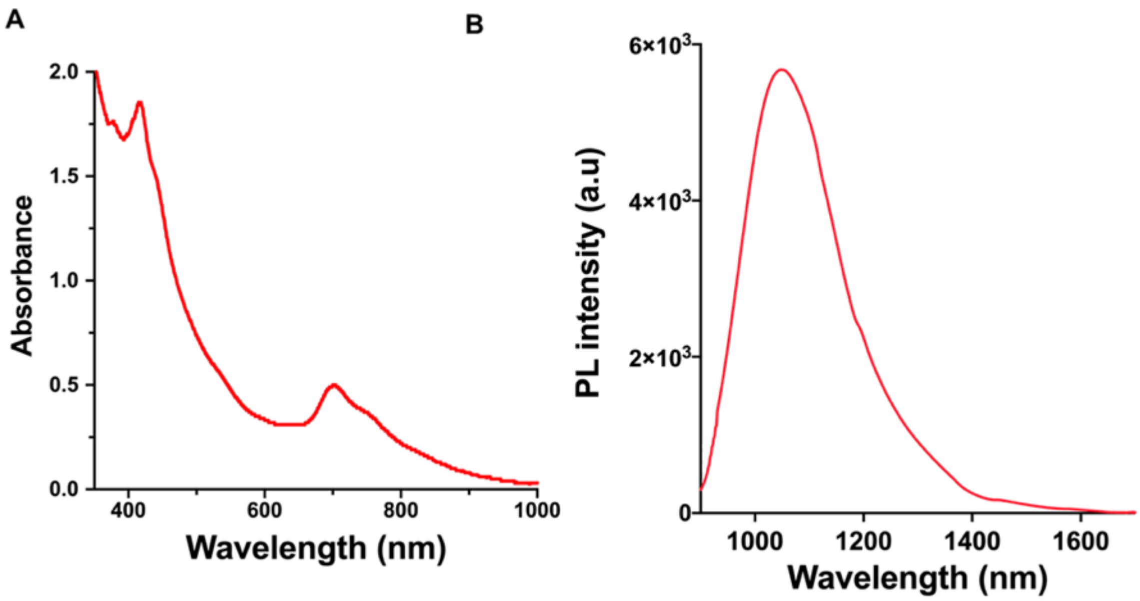

Figure S3. Absorption (A) and PL emission (B) spectra of hydrophobic AuNCs (used in AuPolyNPs) in ethanol under excitation at 808 nm.[4]

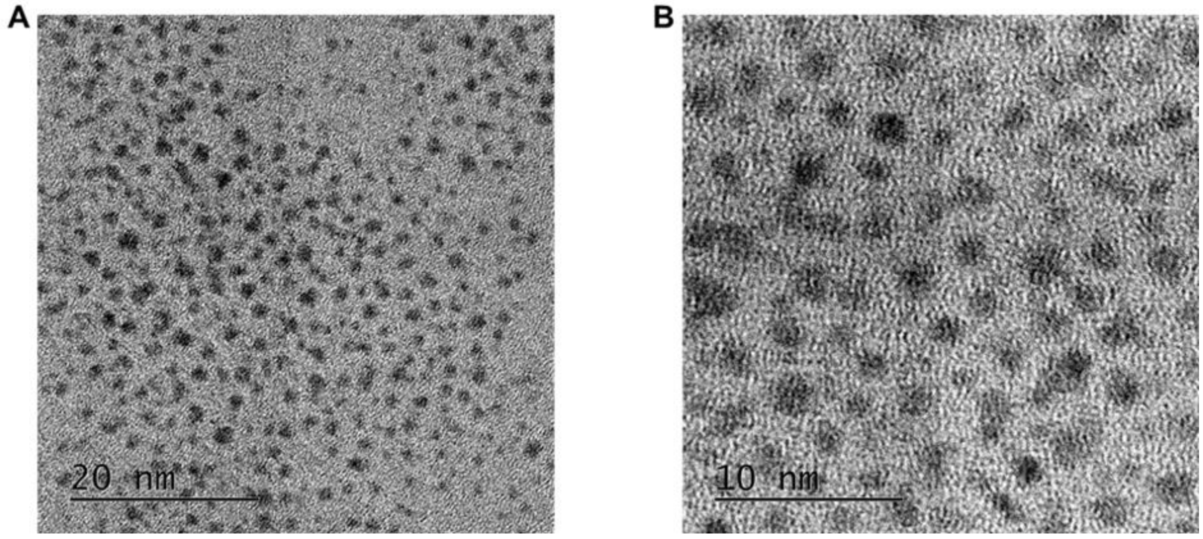

Figure S4. TEM images of hydrophobic AuNCs (used in AuPolyNPs) at two different magnifications.

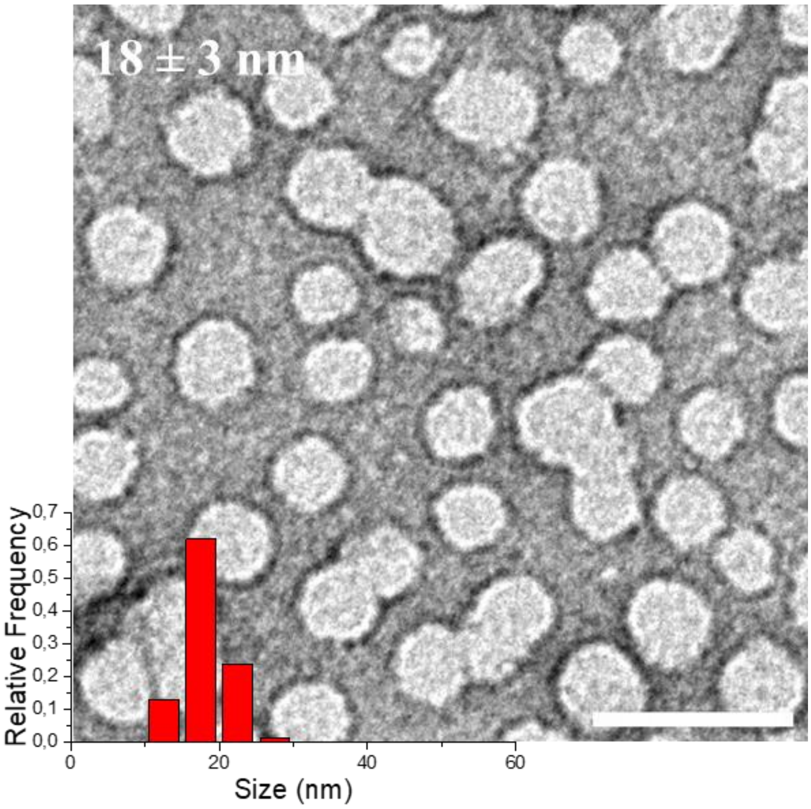

Figure S5. TEM images of negatively stained AuPolyNPs with their relative size frequency (bottom left). Scale bar 50 nm.

Supplementary movie

Movie M1: AuPolyNPs diffusing in a 2:1 v/v glycerol-water mixture imaged under excitation at 660 nm (700W/cm^2, 30ms/frame). Scale Bar 10 µm.

References

[1] X. Le Guével, K. D. Wegner, C. Würth, V. A. Baulin, B. Musnier, V. Josserand, U. Resch-Genger, J. Coll, *Chem. Commun.* **2022**, *58*, 2967.
[2] J. K. Streit, S. M. Bachilo, S. Ghosh, C.-W. Lin, R. B. Weisman, *Nano Lett.* **2014**, *14*, 1530.
[3] X. Wei, T. Tanaka, S. Li, M. Tsuzuki, G. Wang, Z. Yao, L. Li, Y. Yomogida, A. Hirano, H. Liu, H. Kataura, *Nano Lett.* **2020**, *20*, 410.
[4] L. Haye, P. I. Diriwari, A. Alhalabi, T. Gallavardin, A. Combes, A. S. Klymchenko, N. Hildebrandt, X. Le Guével, A. Reisch, *Advanced Optical Materials* **2022**, 2201474.
