# Supplementary figures and images for: Expanding the Palette of SWIR Emitting Nanoparticles Based on Au Nanoclusters for Single‐Particle Tracking Microscopy
Source: Adv Sci (Weinh). 2024 Apr 19;11(24):2309267. doi: 10.1002/advs.202309267 (PMC11199965; doi:10.1002/advs.202309267)

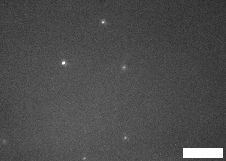

Supplement: Supplementary file 2 — Supplemental Movie 1 [file ADVS-11-2309267-s002.gif]
